# A Feasible Method for Evaluating Post-Stroke Knee Spasticity: Pose-Estimation-Assisted Pendulum Test

**DOI:** 10.3390/life15111760

**Published:** 2025-11-16

**Authors:** Yun-Chien Yeh, Ching-Shiou Tang, Quang Hung Ho, Cheng-Yu Tsai, Jiunn-Horng Kang

**Affiliations:** 1 School of Biomedical Engineering, Taipei Medical University, Taipei 110, Taiwan; 2 International Ph.D. Program in Medicine, Taipei Medical University, Taipei 110, Taiwan; 3 Rehabilitation Department, Cho Ray Hospital, Ho Chi Minh City 700000, Vietnam; 4 Graduate Institute of Nanomedicine and Medical Engineering, College of Biomedical Engineering, Taipei Medical University, Taipei 110, Taiwan; 5 Department of Physical Medicine and Rehabilitation, Taipei Medical University Hospital, Taipei 11031, Taiwan; 6 TMU Research Center of Artificial Intelligence in Medicine and Health, Taipei Medical University, Taipei 110, Taiwan; 7 Department of Physical Medicine and Rehabilitation, School of Medicine, College of Medicine, Taipei Medical University, Taipei 110, Taiwan

**Keywords:** spasticity, stroke, pendulum test, markerless pose estimation, agreement

## Abstract

Purpose: Post-stroke spasticity (PSS) substantially affects functional recovery and quality of life in stroke survivors. However, the current clinical assessment methods exhibit certain subjectivity and equipment limitations. Human pose estimation presents a promising alternative for objective and user-friendly spasticity assessment. Materials and Methods: A total of 20 stroke survivors with PSS underwent pendulum tests with smartphones from multiple angles to quantitatively assess knee muscle spasticity. Pose estimation was conducted using the AlphaPose and STCFormer algorithms, with simultaneous measurements using an electronic goniometer as a reference. Three pendulum parameters were evaluated: normalized relaxation index (P1), first maximum of oscillation (P2), and relaxation index at half swing (P3). Bland–Altman analyses were used to analyze the consistency between pose estimation and electronic goniometer measurements. Intraclass correlation coefficient (ICC) and Spearman’s correlation analyses were conducted to evaluate agreement and reliability between electronic goniometer measurements and clinical evaluation. Results: P1 demonstrated the highest consistency between pose estimation and electronic goniometer measurements, with the highest ICC values (0.931 for AlphaPose and 0.911–0.94 for STCFormer). P1 and P3 differentiated between affected and unaffected limbs (*p* < 0.01) and demonstrated significant negative correlations with Modified Ashworth Scale scores, particularly for knee extensors (P1: ρ = −0.747 for AlphaPose and −0.781 for STCFormer; *p* < 0.01). P2 demonstrated low consistency and differential performance across all analyses. Conclusions: Video-based human pose estimation, particularly using P1, offers a reliable and objective method for evaluating PSS, demonstrating strong agreement with electronic goniometer measurements. This approach is clinically feasible for evaluating spasticity.

## 1. Introduction

Stroke is a major global health concern that ranks third among the leading causes of death and disability worldwide. Over the past 30 years, the number of individuals affected by stroke has nearly doubled, reaching 94 million [1]. Among the many complications of stroke, post-stroke spasticity (PSS) is a common and serious condition that substantially affects patients’ functional recovery. PSS is characterized by velocity-dependent increases in muscle tone and enhanced stretch reflexes resulting from upper motor neuron lesions [2]. This condition may substantially affect patients’ mobility and rehabilitation outcomes, manifesting as abnormal muscle activity and impaired motor control. It may also impact balance and gait recovery, increase fall risk, and ultimately reduce quality of life [3]. Its prevalence varies over time, with rates ranging from 27% at 1 month to 43% at 6 months post-stroke, and joint contractures may develop as early as 3–6 weeks. Given the substantial impact of PSS on functional recovery, early, accurate and objective evaluation of the severity of spasticity is crucial for optimizing clinical treatment and rehabilitation strategies [4,5].

Currently, commonly used clinical methods for evaluating spasticity include the Modified Ashworth Scale (MAS) and the Modified Tardieu Scale (MTS). The MAS evaluates spasticity by manually measuring resistance during passive limb stretching, whereas the MTS quantifies spasticity by monitoring changes in resistance at different stretching speeds [6]. Although these scales have been widely adopted in clinical practice and provide quantitative assessments, their scores are often influenced by subjective judgment, resulting in interrater variability and diminished objectivity. This inherent subjectivity may, in turn, compromise the consistency and reliability of therapeutic decision-making [7,8]. These limitations have driven the development of more objective assessment approaches, including electrophysiological and biomechanical methods that rely on sensors to yield objective results [4]. Among these biomechanical methods, the pendulum test has been commonly used to analyze limb mechanical properties during passive swinging. During the pendulum test, lower-limb swing movement is observed after a free fall under the influence of gravity, which enables the assessment of joint stiffness and muscle tone. This test is effective and feasible because of its simplicity, speed, repeatability, and noninvasiveness [7,9,10,11]. However, traditional implementations require specialized equipment, which limits their clinical application due to resource-intensive installation, high costs, and environmental constraints.

Recent advancements in deep learning have enabled the development of markerless human pose estimation techniques as promising tools for movement evaluation. These techniques have been widely used in clinical assessments and rehabilitation [12]. By relying on computer vision algorithms, markerless human pose estimation techniques enable noninvasive, low-cost, and high-precision kinematic analyses without requiring additional sensors or wearable devices. Hence, integrating image analysis technology with the pendulum test represents a novel approach to spasticity assessment, potentially automating severity assessment while mitigating subjective bias and equipment dependence. We hypothesize that markerless pose estimation can be applied to the pendulum test to derive quantitative parameters for evaluating knee spasticity. Markerless pose estimation could be particularly valuable in resource-limited settings such as rehabilitation units without access to costly equipment and may also enable convenient patient monitoring at home or through telemedicine platforms.

In this study, we aim to integrate video-based human pose estimation technology into the concept of pendulum testing to establish an objective and clinically feasible spasticity assessment. Our primary goal was to confirm whether this approach can achieve comparable accuracy to existing methods while distinguishing between affected and unaffected limbs, correlating with MAS assessments, and providing a simple and reliable tool for PSS assessment.

## 2. Methods

### 2.1. Participants

A total of 20 stroke survivors with PSS were recruited from Taipei Medical University Hospital. Although the limited sample size is suitable for a proof-of-concept investigation, it constrains the broader applicability and generalizability of the findings. All participants presented with spasticity of flexor or extensor muscles crossing the knee joint, as measured by the MAS and assessed by an experienced physician. The inclusion criteria were the following: (1) being between 20 and 75 years of age, (2) having received a diagnosis of stroke resulting in spasticity, (3) being able to sit independently, and (4) being able to communicate in Mandarin or Taiwanese. The exclusion criteria were as follows: (1) having joint instability or major pain in the lower limbs or currently using any internal or external fixation devices around the knee joint or (2) having any deformity or amputation in the lower limbs. Rehabilitation programs and anti-spasticity treatments were permitted for all participants. All participants provided written informed consent before enrollment. This study was approved by the Joint Institutional Review Board of Taipei Medical University (approval no. N202211040). To administer the MAS, each participant was asked to assume a supine position, and then an experienced physician passively moved their knee through its full range of motion at a constant speed of approximately 1 s per movement. During this passive stretch, the physician evaluated the resistance encountered and assigned a score from 0 to 4 on the basis of the MAS criteria, with a higher score indicating greater spasticity [13].

### 2.2. Pendulum Test Protocol

**Figure 1:**
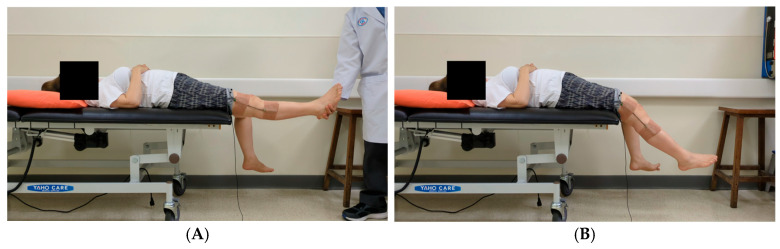

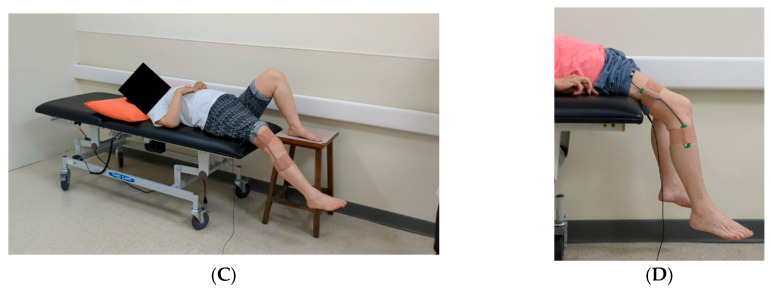
Experimental setup for pendulum testing. (**A**) In the standard testing position, the nontest limb should remain relaxed and should hang freely over the edge of the bed. The figure depicts the camera angles used to enhance depth perception for improving the performance of the three-dimensional human pose estimation model. (**B**) Oblique view captured at 45° above the horizontal plane. (**C**) Lateral view captured at a direct side angle. (**D**) An electronic goniometer is used to measure angles in the pendulum test.

Figure 1 depicts the experimental setup. Each participant underwent standardized pendulum testing on their affected and unaffected limbs, with both full knee extension and relaxation. Each participant was asked to assume a supine position on a bed with their test leg extended horizontally. Each limb was allowed to fall and swing freely under the influence of gravity after being released by the examiner. Five tests were conducted for each limb, resulting in a total of 10 tests per participant. All tests were conducted by experienced clinicians and were video-recorded with two smartphones. Measurements were also conducted using an electronic goniometer. These smartphones were mounted on tripods at the same height and distance, positioned to capture both lateral views and 45° oblique angles. This setup enabled full-body and depth capture for human pose estimation. The field of view was adjusted to ensure that the entire lower limbs and pelvic region remained visible throughout the testing procedure.

### 2.3. Measurement Systems

An electronic goniometer equipped with a ProComp Infiniti eight-channel biofeedback system (Thought Technology, Montreal, QC, Canada) was used to measure joint angles. This goniometer had two sensors: a proximal sensor and a distal sensor. The proximal sensor was placed 15 cm above the lateral femoral epicondyle, aligned with the femoral axis, whereas the distal sensor was positioned 15 cm below the lateral femoral epicondyle, aligned with the fibular head (Figure 1D). The system was calibrated before each testing session, and data were recorded using Biograph Infiniti 5.1 software.

Two human pose estimation frameworks were used to conduct a comprehensive motion analysis. We employed AlphaPose (2D model) and STCFormer (3D model) to estimate joint angle variations to compare the differences between 2D and 3D pose estimation models in capturing sagittal plane knee joint angles. This analysis also allowed us to evaluate how different video capture conditions may affect the accuracy and reliability of angle estimation. In AlphaPose (two-dimensional, version 0.6.0), a regional multiperson pose estimation framework was implemented. This framework utilized symmetric integral key point regression for key point localization, parametric pose nonmaximum suppression for duplicate elimination, part-guided proposal generation for model robustness, and pose-aware identity embedding for temporal consistency [14,15,16]. In STCFormer (three-dimensional, version 2023), a spatiotemporal crisscross attention transformer was implemented. This transformer combined temporal attention for tracking joint movement over time with spatial attention for analyzing joint structure per frame through a parallel, two-pathway attention block. This system incorporated structure-enhanced positional embedding to integrate human skeleton topology and local motion patterns [17]. Both frameworks were deployed in Python environments with graphics processing unit acceleration on a GeForce RTX 4090 graphics card supporting AlphaPose and STCFormer. Although AlphaPose and STCFormer were developed using general-purpose datasets such as COCO and Human3.6M, adapting these models to clinical contexts, particularly those involving supine positions, presents significant domain adaptation challenges.

### 2.4. Data Analysis

After participants were recruited, human pose estimation models were applied to recorded videos. AlphaPose provided two-dimensional coordinates with confidence scores for 17 key points, whereas STCFormer generated three-dimensional spatial coordinates for the same landmarks. Both models extracted data at 30 frames per second. The vectors used to compute joint angles were constructed using the estimated anatomical joint centers derived from pose estimation key points (i.e., hip, knee, and ankle). These do not represent true segment centers of mass, but rather surface-level approximations of joint locations as defined by the pose estimation models. The knee joint angle was calculated using the vector relationships between hip, knee, and ankle points.

For AlphaPose, knee angle was calculated using the following dot product formula:

$$cos\;\theta \;=\frac{\vec{u\;}\;\cdot \;\vec{v\;}}{\left|\vec{u\;}\right|\;\left|\vec{v\;}\right|}$$

$$\theta ={cos}^{-1}\;\frac{\vec{u\;}\;\cdot \;\vec{v\;}}{\left|\vec{u\;}\right|\;\left|\vec{v\;}\right|}$$

where $\vec{u}$ represents the vector from the key point of the hip to the key point of the knee and $\vec{v}$ represents the vector from the key point of the knee to the key point of the ankle as extracted from the pose estimation output.

For STCFormer, the sagittal plane was first defined using vectors from the body midline, denoted as $\vec{u_{m}}$ and $\vec{v_{m}}$. The normal vector $\vec{n}$ to this plane was calculated as

$$\vec{n}=\vec{u_{m}}\times \vec{v_{m}}$$

For each limb, we calculated the components of the hip-to-knee vector $\vec{u}\;$ and knee-to-ankle vector $\vec{v}$ that are perpendicular to the sagittal plane:

$$\vec{u_{v}}\;={proj}_{\vec{n}}\;(\vec{u})\;=\;\frac{\vec{u}\;\cdot \;\vec{n}}{{\left|\left|\vec{n}\right|\right|}^{2}}\;\vec{n}$$

$$\vec{v_{v}}={proj}_{\vec{n}}\;(\vec{v})=\frac{\vec{v}\;\cdot \;\vec{n}}{{\left|\left|\vec{n}\right|\right|}^{2}}\;\vec{n}$$

We then determined the components of these vectors that lie within the sagittal plane:

$$\vec{u_{h}}\;=\;\vec{u}\;-\;\vec{u_{v}}\;=\vec{u}\;-\;\frac{\vec{u}\;\cdot \;\vec{n}}{{\left|\left|\vec{n}\right|\right|}^{2}}\;\vec{n}$$

$$\vec{v_{h}}=v-\vec{v_{v}}=\vec{v}-\frac{\vec{u}\;\cdot \;\vec{n}}{{\left|\left|\vec{n}\right|\right|}^{2}}\;\vec{n}$$

Finally, we calculated the angle between these projected vectors:

$$cos\;\theta \;=\frac{\vec{u_{h}\;}\;\cdot \;\vec{v_{h}\;}}{\left|\vec{u_{h}}\right|\;\left|\vec{v_{h}}\right|}$$

$$\theta ={cos}^{-1}\;(\frac{\vec{u_{h}\;}\;\cdot \;\vec{v_{h}\;}}{\left|\vec{u_{h}}\right|\;\left|\vec{v_{h}}\right|})$$

To enhance the quality of the knee angle data, we implemented a third-order Savitzky–Golay filter for signal smoothing. This filter effectively reduced noise from minor body movements and mitigated measurement fluctuations while preserving key waveform characteristics, particularly the peaks and troughs essential for parameter identification.

**Figure 2:**
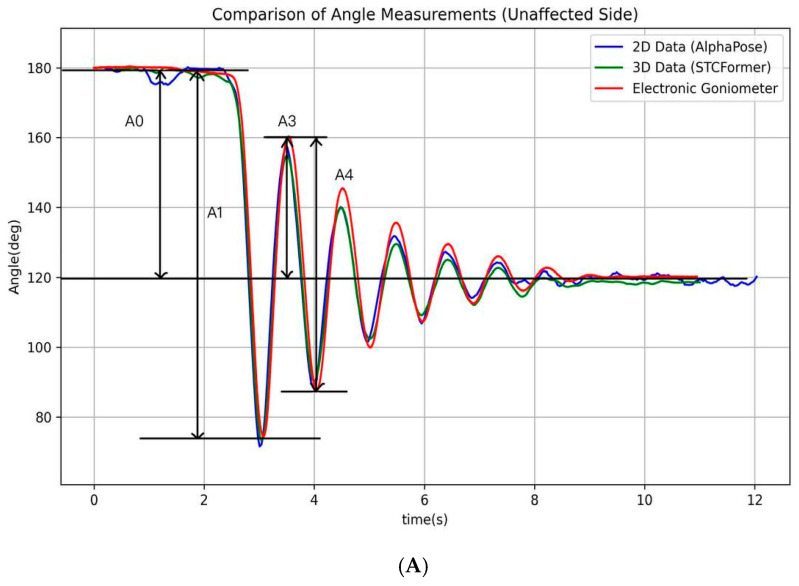

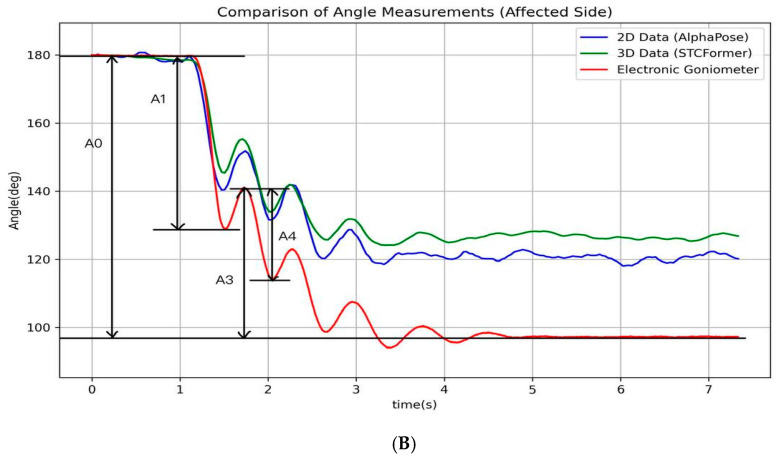
The illustration of the angle–time curves of a typical pendulum test. The figure depicts the angle–time curves recorded by an electronic goniometer and calculated using the AlphaPose and STCFormer human pose estimation models for the affected and unaffected sides during a typical pendulum test. (**A**) Unaffected side; (**B**) affected side.

**Figure 3:**
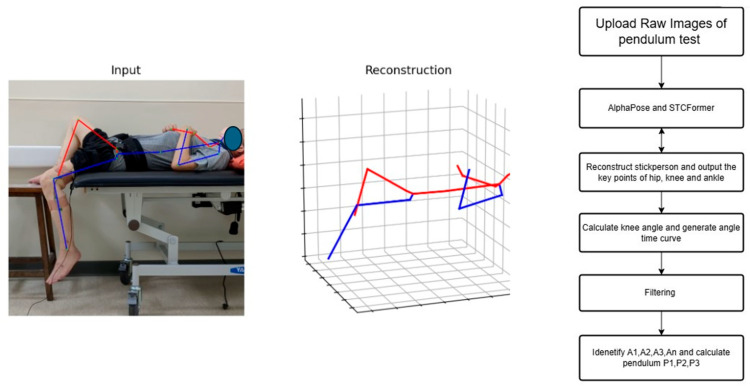
The analysis workflow and schema.

Pendulum parameters were derived from the angle–time curve (Figure 2) through key positions identified by locating the local maximum and minimum of the curve. These key points (A_0_, A_1_, A_3_, and A_4_) were then used to calculate the following clinically relevant pendulum parameters:

$$P1\;(\mathrm{normalized}\;\mathrm{relaxation}\;\mathrm{index})\;=\;\frac{A_{1}}{A_{0}}$$

$$P2\;(\mathrm{first}\;\mathrm{maximum}\;\mathrm{of}\;\mathrm{oscillation})=A_{3}$$

$$P3\;(\mathrm{relaxation}\;\mathrm{index}\;\mathrm{at}\;\mathrm{half}\;\mathrm{swing})=\frac{A_{4}}{A_{3}}$$

where A_0_ represents the angular difference between the raised initial position and the natural resting position of the knee, establishing the baseline range of movement. During the first swing, A_1_ captures the maximum angular change, measured from the starting position to the lowest point of the first swing, establishing the initial motion amplitude. During the second swing, A_3_ captures the angular change between the peak of the second swing and the resting position, which helps evaluate energy retention in the joint during continuous motion. Finally, A_4_ captures the maximum angular change of the second swing cycle, from its highest to lowest point, demonstrating how the joint continues to move after the initial momentum has partially dissipated [18,19]. Biomechanically, P1 reflects the degree of movement inhibition due to muscle tone, P2 indicates the initial freedom of movement, and P3 serves as an indicator of energy loss in subsequent oscillatory cycles. Together, these measurements provide a comprehensive assessment of knee joint kinematics. All processing procedures were performed using Python 3.10. The analytical procedure and its schematic representation are presented in Figure 3. The pseudocode in this study is provided in the Supplementary Materials.

### 2.5. Statistical Analysis

To validate the consistency of data between the different measurement methods, Bland–Altman plot analyses were conducted to assess the agreement between the video-based recognition system and the electronic goniometer system. This analysis included the calculation of mean differences and 95% limits of agreement, providing a visual representation of measurement consistency to detect systemic bias. To examine the presence of proportional bias, linear regression analysis was performed with the mean of the two measurements as the independent variable and their difference as the dependent variable.

To further quantify consistency, continuous measurement values from both systems were subjected to an intraclass correlation coefficient (ICC) analysis to measure the degree of agreement for continuous data. In this study, the ICC was calculated using a two-way random-effects model (ICC (2,1)) with a consistency type. The ICC can be interpreted as follows: values below 0.5 indicate poor reliability, between 0.5 and 0.75 indicate moderate reliability, between 0.75 and 0.9 indicate good reliability, and above 0.9 indicate excellent reliability [20]. To determine each system’s ability to distinguish between affected and unaffected limbs, paired *t*-tests were conducted to compare the pendulum parameters in the bilateral limbs of the same participants. Cohen’s d was calculated to quantify the effect size, indicating the magnitude and direction of differences between the affected and unaffected limbs, with small, medium, and large effects corresponding to 0.1, 0.4, and 0.8, respectively [21].

Spearman’s correlation analysis was then used to evaluate the monotonic relationships between these pendulum parameters and MAS clinical scores. Spearman’s correlation coefficients were interpreted based on effect size thresholds, with r values of 0.3, 0.5, and 0.6 indicating small, medium, and large effects, respectively [21]. Before conducting the correlation analysis, outliers likely resulting from inadequate muscle relaxation were identified and removed using the interquartile range method, excluding approximately 1.5% of the total dataset.

Prior to conducting parametric analyses, the normality of the differences between measurement methods was assessed using the Shapiro–Wilk test. This procedure was applied to verify that the data met the assumption of normality required for subsequent analyses, including paired *t*-tests and Bland–Altman assessments. All statistical analyses were conducted using Python 3.10, with statistical significance set at α = 0.05.

## 3. Results

Patient Characteristics. A total of 20 patients with stroke (12 men and 8 women, mean age: 59.6 years, with standard deviation (SD): 9.3) were recruited. We list these patients’ clinical data and MAS scores for knee flexors and extensors in Supplementary Table S1.

**Figure 4:**
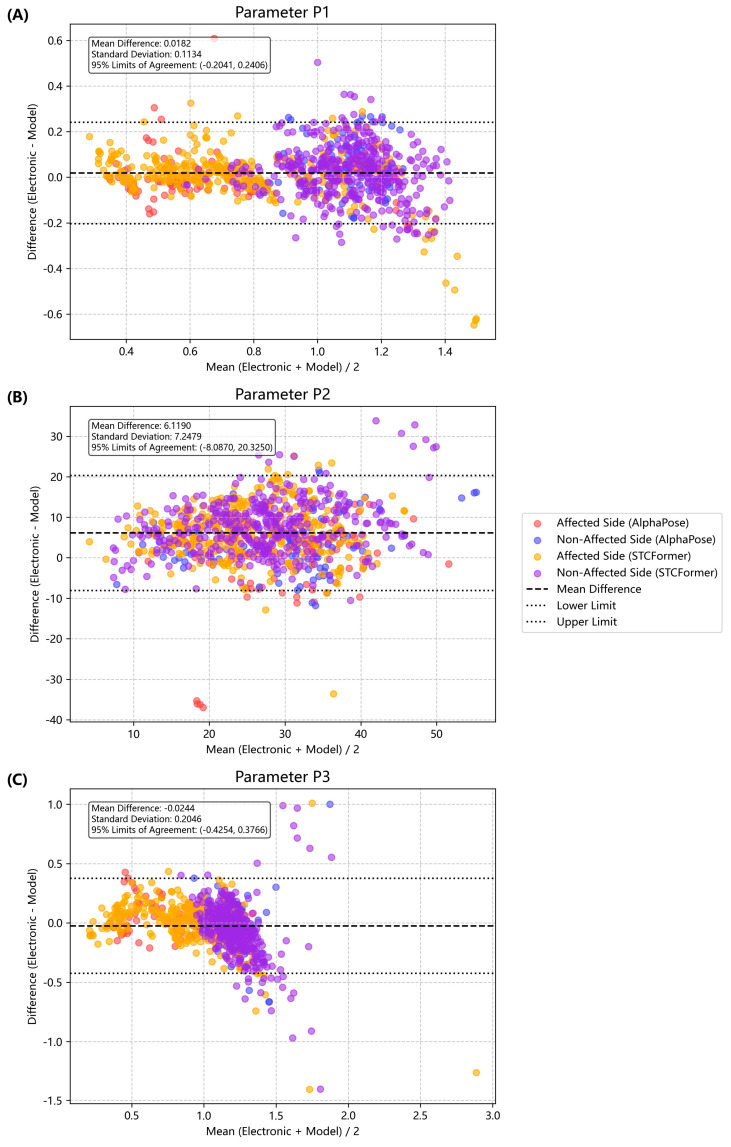
Bland–Altman plots for pendulum parameters. Each point represents an individual measurement (red O: affected side, AlphaPose; blue O: unaffected side, AlphaPose; yellow X: affected side, STCFormer; and purple X: unaffected side, STCFormer). (**A**) Comparison of the normalized relaxation index (P1). (**B**) Comparison for the first maximum of oscillation (P2) (difference in degrees). (**C**) Comparison for the relaxation index at half swing (P3).

Agreement Analyses. Among these three parameters, P1 consistently demonstrated the smallest bias and narrowest limits of agreement across both AlphaPose and STCFormer models (Figure 4). Compared with P1, P2 demonstrated higher mean differences (up to 9.0° in STCFormer), indicating notable systematic deviations. P3 remained moderately stable but less consistent than P1. We found no proportional bias between the AlphaPose and electronic goniometer measurements. However, we found no proportional bias between the AlphaPose and electronic goniometer measurements. However, mild proportional bias was observed in the STCFormer measurements, specifically for P2 on the non-affected side and P3 on the affected side, when compared with the goniometer data (R^2^ = 0.29, *p* = 0.0167 & R^2^ = 0.26, *p* = 0.0216, respectively) (Supplementary Table S2).

The ICC analysis revealed that AlphaPose demonstrated higher agreement with the electronic goniometer compared to STCFormer across most pendulum parameters (Table 1). For AlphaPose, excellent reliability was observed for P1 and P3 in the affected limbs (ICC = 0.93 & 0.892), and good overall agreement, indicating stable performance in capturing major pendulum motion patterns. In addition, STCFormer showed slightly lower ICCs overall (0.917 & 0.761 for P1 and P3, respectively) and greater variability across viewing angles, particularly in P2 and the unaffected limbs. These findings suggest that, under controlled recording conditions, the 2D AlphaPose system can achieve robust agreement with gold-standard goniometer data to measure P1 and P3 on the affected limbs, whereas STCFormer may require further optimization to improve consistency. Human pose estimation methods typically show lower agreement for P2 measurements, potentially due to greater variation in pendulum dynamics during measurement.

Discriminative Validity. The distribution of pendulum data fits a normal distribution according to the Shapiro-Wilk test (Supplementary Table S3). We found, across all measurement methods, P1 and P3 showed significant differences between the affected and unaffected limbs, with moderate effect sizes (|d| ≈ 0.35–0.37). By contrast, P2 exhibited inconsistent trends across methods. The electronic goniometer, STCFormer, and AlphaPose did not reach statistical significance (*p* = 0.2456). The effect size of P2 varied widely across methods, suggesting that this parameter may be more sensitive to measurement variability or differences in model estimation (Table 2). Overall, the findings indicate that P1 and P3 are reliable indicators of altered pendulum dynamics in the affected limb, while P2 may be influenced by factors beyond spasticity, such as voluntary damping or passive stiffness.

Clinical Correlations. We found that the electronic goniometer demonstrated significant negative associations with medium to large effect sizes between MAS scores and pendulum parameters (P1 & P3) (ρ ranging from −0.443 to −0.773, *p* < 0.0001), confirming that higher spasticity corresponds to smaller oscillation amplitudes (Table 3). Among the markerless systems, the STCFormer model showed correlation patterns closely matching the goniometer, with significantly negative correlations in both knee flexors and extensors (ρ ≈ −0.452 to −0.781, *p* < 0.0001). In contrast, AlphaPose showed less consistent correlations, particularly in the knee flexor measurements, suggesting that the 2D estimation approach is limited in capturing subtle movement constraints. Across all systems, P1 and P3 parameters showed the highest agreement with MAS scores, indicating their robustness as clinical indicators of spasticity severity, whereas P2 exhibited greater variability and lower sensitivity.

## 4. Discussion

In this study, the results of human posture estimation were found to be highly consistent with electronic goniometer measurements across different parameters. P1 demonstrated the highest consistency in Bland–Altman plot analyses without clear bias. It also demonstrated optimal ICC performance on the affected side, with a high correlation coefficient (>0.93). P2 demonstrated significant systematic bias with large mean differences and standard deviations, whereas P3 maintained moderate stability with a correlation coefficient of >0.85. This high performance of P1 and P3 as ratio parameters likely originated from their ability to mitigate systematic measurement errors. However, its robustness warrants further validation in larger, more heterogeneous stroke populations to ensure generalizability and clinical reliability.

P1 and P3 effectively differentiated between the affected and unaffected legs (*p* < 0.01), with performance comparable to that of the electronic goniometer. By contrast, P2 failed to achieve significance (*p* > 0.05). The effectiveness of P1 and P3 likely reflected their sensitivity to changes in muscle tone characteristic of spasticity. The focus of P1 on the initial-to-total swing ratio directly captured movement resistance effects, whereas P2 may have been susceptible to other factors, which reduced its specificity for spasticity detection. These results are consistent with Yeh et al. [18], who reported that both electronic-goniometer-measured and Wii-measured P1 and P3 effectively identified spasticity. Meanwhile, these findings are similar to those of Katz et al. [9], who reported that the corrected relaxation index (equivalent to P1) performed well in spasticity identification. In the present study, P1 and P3 in both systems demonstrated significantly negative correlations with MAS scores (*p* < 0.01). By contrast, P2 demonstrated weaker correlations, especially for knee extensors (*p* > 0.05). These strong correlations between P1 and P3 likely reflected their ability to capture increased muscle tone and hyperactive tendon reflexes, key features of spasticity. This negative correlation indicated that greater spasticity severity (higher MAS scores) corresponded to lower parameter values, indicating movement restriction and enhanced muscle tone.

The stability of P1 and P3 parameters may stem from their calculation method: they are standardized indicators calculated as ratios of joint angles, effectively offsetting baseline differences caused by posture changes and reducing the influence of individual differences, instrument setup, or reference system selection. In contrast, P2 directly measures the maximum swing, making it more susceptible to changes in posture and other external factors. Rahimi’s research shows that ratio parameters in pendulum tests are more stable than absolute angle parameters and are less likely to be affected by test conditions [10]. Given these findings, we recommend prioritizing P1 as the primary indicator for spasticity assessment using markerless video recordings.

A stronger correlation was observed with knee extensors than with knee flexors, consistent with the pendulum test’s primary assessment of extensor stretch responses [22]. Despite the limitations of the MAS in terms of subjectivity and variations in interrater reliability among health-care professionals, the MAS remains the most widely used clinical spasticity assessment tool [23]. It is worth noting that the MAS primarily reflects the subjective perception of resistance during passive stretching, whereas the pendulum test captures dynamic, quantitative indicators during movement. Therefore, the differences in their correlations are not merely technical but also arise from the distinct physiological phenomena each method assesses. Combining static MAS assessments with dynamic pendulum testing may enable more comprehensive spasticity evaluation, as suggested by systematic reviews that found pendulum tests capture increases in velocity-dependent muscle tone that MAS cannot [11].

In this study, a significant correlation was observed between P1 or P3 and MAS scores, indicating that video-based markerless systems can capture clinically relevant spasticity features. This approach offers advantages, including simple settings and minimal equipment requirements, and it facilitates objective quantification to complement subjective clinical assessments. Hence, evidence supports markerless pendulum testing as a feasible solution for standardized spasticity evaluation in clinical practice.

This study has several limitations. First, this study’s small sample size significantly limits the generalizability of its findings. The limited number of cases fails to comprehensively reflect characteristics of varying degrees of spasticity and different patient groups, increasing the risk of Type II errors and reducing statistical power, potentially masking subtle but clinically meaningful differences between measurement methods. For instance, the study mostly included participants with Modified Ashworth Scale (MAS) scores ranging from 0 to 2, while excluding patients with more severe spasticity (MAS 3–4), causing the research results to incompletely represent the full spectrum of PSS severity. Furthermore, the sample size limitation affects the assessment of the system’s adaptability across different ages and genders, disease severity, and various environmental conditions. Secondly, the MAS measurement conducted in this study is subjective and may not have fully captured the velocity-dependent characteristics. Further validation against the MTS and other biomechanical ground-truth measures is advised. Thirdly, the human pose estimation algorithms used in this study were optimized for daily activities rather than for tasks in the supine position [24,25], resulting in reduced accuracy during rapid pendulum movements. Fourth, validation of the approach in a more diverse patient cohort, including individuals with spasticity from various etiologies such as spinal cord injury and multiple sclerosis, is warranted to confirm its generalizability. Finally, our study was conducted in a controlled environment; however, variations in camera positions, lighting conditions, occlusion, and background commonly encountered in real-world conditions may compromise the performance of markerless motion capture systems.

Future work should consider addressing (i) methodological improvements: incorporating alternative spasticity assessment tools, such as the MTS, would facilitate the comprehensive validation of velocity-dependent spastic characteristics and enhance clinical applicability. (ii) Technical improvements: various emerging clinical pose estimation models, such as PatientPose, have demonstrated promise in implementation scenarios involving scene lighting standardization techniques and patient-specific convolutional neural networks, achieving improvements of up to 42.4% in accuracy compared with general-purpose algorithms [26]. Integrating advanced temporal filtering methods and multicamera systems may further address motion blur and occlusion challenges inherent in rapid pendulum movements. In addition, developing specialized datasets focused on clinical postures and movement patterns, combined with advanced data augmentation techniques such as PoseAug [27], may increase model adaptability across diverse clinical scenarios. (iii) Clinical translation: integrating spatiotemporal dynamic characteristics into pose estimation models and adopting multiangle photography or depth camera technology may enhance the accuracy of three-dimensional joint position reconstruction [28]. Overall, optimizing system interface design and operational processes is essential for enhancing clinical adaptability, particularly in resource-limited environments in which simplified operational steps and reduced environmental requirements are essential. These technological advancements, combined with additional clinical validation studies, would establish pose-estimation-based spasticity assessment as a reliable, objective, and accessible tool for neurological rehabilitation practice.

## 5. Conclusions

Video-based human pose estimation enables a feasible, objective assessment of knee muscle spasticity in patients with stroke. P1 is a robust parameter that demonstrates excellent agreement with electronic goniometer measurements. This markerless approach facilitates simple, cost-effective, and feasible spasticity evaluation in clinical settings.

## Figures and Tables

**Table 1:** ICC agreement analysis between human pose estimation and electronic goniometer measurements.

Measurement	Category	Pendulum Parameters from Electronic Goniometer		
		P1 (95% CI)	P2 (95% CI)	P3 (95% CI)
AlphaPose	Affected	0.931 (0.9, 0.95)	0.468 (0.3, 0.61)	0.892 (0.84, 0.93)
	Unaffected	0.553 (0.37, 0.69)	0.727 (0.49, 0.84)	0.199 (0, 0.38)
	Overall	0.929 (0.91, 0.95)	0.613 (0.51, 0.7)	0.808 (0.75, 0.85)
STCFormer	Affected (45°)	0.911 (0.88, 0.93)	0.449 (−0.1, 0.75)	0.853 (0.81, 0.89)
	Affected (90°)	0.939 (0.87, 0.97)	0.653 (0.3, 0.81)	0.823 (0.77, 0.86)
	Unaffected (45°)	0.77 (0.68, 0.83)	0.521 (−0.09, 0.8)	0.132 (0, 0.26)
	Unaffected (90°)	0.659 (0.24, 0.82)	0.619 (0.09, 0.82)	0.341 (0.21, 0.46)
	Overall	0.917 (0.9, 0.93)	0.56 (−0.01, 0.79)	0.761 (0.73, 0.79)

Intraclass correlation coefficient (ICC) analysis of agreement between markerless human pose estimation models and electronic goniometer measurements for pendulum parameters (P1, P2, and P3). Values are presented as ICC (95% confidence interval). A two-way random-effects model [ICC(2,1), consistency type] was used to assess agreement between measurement methods.

**Table 2:** Paired *t*-test analysis results comparing pendulum parameters in the affected and unaffected limbs.

Measurement	Value	Pendulum Parameters		
		P1	P2	P3
**Electronic** **goniometer**	Affected side Unaffected side Effect size	0.74 (0.26) 1.11 (0.14) −0.37	28.4 (7.98) 32.9 (9.4) −4.49	0.87 (0.27) 1.18 (0.12) −0.31
	*p* value	<0.0001 *	0.0892	0.0003 *
AlphaPose	Affected side Unaffected side Effect size	0.69 (0.23) 1.06 (0.1) −0.37	27.2 (6.57) 28.7 (8.48) −1.49	0.87 (0.27) 1.18 (0.18) −0.31
	*p* value	<0.0001 *	0.606	0.0017 *
STCFormer	Affected side Unaffected side Effect size	0.73 (0.29) 1.07 (0.13) −0.34	21.7 (6.94) 24.0 (6.57) −2.34	0.87 (0.3) 1.23 (0.14) −0.35
	*p* value	<0.0001 *	0.28	<0.0001 *

Paired *t*-test results comparing pendulum parameters (P1, P2, and P3) between the affected and unaffected limbs measured by the electronic goniometer, AlphaPose, and STCFormer models. Values are presented as the mean ± standard deviation (SD). Cohen’s d indicates the effect size, with negative values reflecting lower parameter values in the affected limb. Asterisks (*) denote statistically significant differences (*p* < 0.05).

**Table 3:** Spearman’s correlation analysis results of pendulum parameters and MAS scores.

Measurement	MAS	Value	Pendulum Parameters		
			P1	P2	P3
**Electronic** **goniometers**	Knee flexors	ρ	−0.51	−0.443	−0.446
		*p* value	<0.0001 *	<0.0001 *	<0.0001 *
	Knee extensors	ρ	−0.773	−0.188	−0.746
		*p* value	<0.0001 *	0.119	<0.0001 *
AlphaPose	Knee flexors	ρ	−0.287	0.041	−0.246
		*p* value	0.286	0.880	0.358
	Knee extensors	ρ	−0.747	0.268	−0.793
		*p* value	<0.0001 *	0.316	<0.0001 *
STCFormer	Knee flexors	ρ	−0.487	−0.292	−0.452
		*p* value	<0.0001 *	0.012	<0.0001 *
	Knee extensors	ρ	−0.781	−0.126	−0.786
		*p* value	<0.0001 *	0.285	<0.0001 *

A negative correlation was observed between pendulum parameters and MAS scores. P1 consistently demonstrated significantly negative correlations with MAS scores, especially for knee extensors. Asterisks (*) denote statistically significant differences (*p* < 0.05).

## Data Availability

Due to institutional review board (IRB) restrictions and patient privacy considerations, the individual-level clinical data cannot be shared publicly. De-identified data may be made available upon reasonable request to the corresponding author and with appropriate approvals.

## Supplementary Materials

The following supporting information can be downloaded at https://www.mdpi.com/article/10.3390/life15111760/s1. Table S1: Clinical information and demographic data of the participants; Table S2: Proportional bias analysis for pose-estimation models versus the electronic goniometer measurements; Table S3: Shapiro-Wilk test for testing normal distribution for pendulum data; 2. Pseudo code of experiment; 3. Python packages and version.
