# Supplementary material for: A Feasible Method for Evaluating Post-Stroke Knee Spasticity: Pose-Estimation-Assisted Pendulum Test
Source: Life (Basel). 2025 Nov 16;15(11):1760. doi: 10.3390/life15111760 (PMC12653666; doi:10.3390/life15111760)
Supplement: Supplementary file 1 [file life-15-01760-s001.zip › life-3887872-supplementary.pdf]

Supplementary Material

1. Tables

Table S1. Clinical information and demographic data of the participants.

Participant no.	Sex	Age	Affected side	MAS score	
				Knee flexors	Knee extensors
1	F	52	L	2	0
2	M	55	R	1	1
3	M	57	L	1+	1
4	F	48	L	1+	2
5	M	51	R	1+	1
6	M	59	L	1+	2
7	F	62	R	1+	2
8	M	50	L	1+	2
9	M	69	L	1+	1
10	F	45	R	1+	2
11	M	54	R	1+	1+
12	M	60	R	1	1
13	M	52	R	1+	1+
14	F	62	R	1+	2
15	M	59	R	1+	1
16	F	79	R	1+	1+
17	M	61	L	1	1
18	M	78	L	1+	2
19	M	-72	R	1+	2
20	M	55	R	1+	2

Abbreviations: M, male; F, female; L, left; R, right; MAS, Modified Ashworth Scale.

Table S2. Proportional bias analysis for pose-estimation models versus the electronic goniometer measurements.

P1: AlphaPose vs. electronic goniometer	Non-affected side	Affected side
Proportional bias (slope)	Estimate = -0.03 (p = 0.943)	Estimate = -0.03 (p = 0.666)
Mean (constant) bias	Intercept = -0.01 (p = 0.981)	Intercept = -0.02 (p = 0.731)
Spread/Limits of Agreement (LoA)	Residual SD = 0.12<br>LoA = [-0.238, 0.220]	Residual SD = 0.06<br>LoA = [-0.127, 0.095]
Model fit	$R^2 = 0.0004$	$R^2 = 0.01$

P2: AlphaPose vs. electronic goniometer	Non-affected side	Affected side
Proportional bias (slope)	Estimate = -0.33 (p = 0.0897)	Estimate = -0.18 (p = 0.550)
Mean (constant) bias	Intercept = -5.99 (p = 0.3162)	Intercept = 3.74 (p = 0.653)
Spread/Limits of Agreement (LoA)	Residual SD = 6.56<br>LoA = [-18.85, 6.85]	Residual SD = 6.87<br>LoA = [-9.73, 17.21]
Model fit	$R^2 = 0.19$	$R^2 = 0.03$

P3: AlphaPose vs. electronic goniometer	Non-affected side	Affected side
Proportional bias (slope)	Estimate = 0.80 (p = 0.0812)	Estimate = 0.17 (p = 0.0452)
Mean (constant) bias	Intercept = -0.94 (p = 0.0825)	Intercept = -0.16 (p = 0.0375)
Spread/Limits of Agreement (LoA)	Residual SD = 0.18<br>LoA = [-1.29, -0.59]	Residual SD = 0.07<br>LoA = [-0.29, -0.02]
Model fit	$R^2 = 0.2$	$R^2 = 0.26$

P1: STCFormer vs. electronic goniometer	Non-affected side	Affected side
Proportional bias (slope)	Estimate = -0.05 (p = 0.776)	Estimate = -0.09 (p = 0.107)
Mean (constant) bias	Intercept = -0.02 (p = 0.901)	Intercept = -0.07 (p = 0.077)
Spread/Limits of Agreement (LoA)	Residual SD = 0.09<br>LoA = [-0.15, 0.19]	Residual SD = 0.06<br>LoA = [-0.19, 0.04]
Model fit	$R^2 = 0.08$	$R^2 = 0.06$

P2: STCFormer vs. electronic goniometer	Non-affected side	Affected side
Proportional bias (slope)	Estimate = -0.41 (p = 0.0167)	Estimate = -0.11 (p = 0.468)
Mean (constant) bias	Intercept = 2.59 (p = 0.5712)	Intercept = -3.87 (p = 0.345)
Spread/Limits of Agreement (LoA)	Residual SD = 4.89<br>LoA = [-6.9, 12.19]	Residual SD = 4.74<br>LoA = [-13.16, 5.42]
Model fit	$R^2 = 0.29$	$R^2 = 0.03$

P3: STCFormer vs. electronic goniometer	Non-affected side	Affected side
Proportional bias (slope)	Estimate = 0.08 (p = 0.771)	Estimate = 0.16 (p = 0.0216)
Mean (constant) bias	Intercept = -0.039 (p = 0.906)	Intercept = -0.14 (p = 0.0284)
Spread/Limits of Agreement (LoA)	Residual SD = 0.12<br>LoA = [-0.28, 0.2]	Residual SD = 0.08<br>LoA = [-0.29, 0.01]
Model fit	$R^2 = 0.005$	$R^2 = 0.26$

For each parameter and limb side, the estimated regression slope and intercept indicate the presence of proportional or constant bias, while the residual standard deviation and limits of agreement (LoA) describe the spread of differences. The R^2 values represent the degree of model fit.

Table S3: Shapiro–Wilk test for testing the normal distribution of pendulum data.

Measurement		Pendulum parameters		
		**P1**	**P2**	**P3**
**Electronic goniometer**	Affected side	p-value = 0.1292	p-value = 0.751	p-value = 0.4979
	Unaffected side	p-value = 0.9049	p-value = 0.4054	p-value = 0.0350
**AlphaPose**	Affected side	p-value = 0.0932	p-value = 0.8495	p-value = 0.2201
	Unaffected side	p-value = 0.9407	p-value = 0.9124	p-value = 0.4909
**STCFormer**	Affected side	p-value = 0.0866	p-value = 0.5928	p-value = 0.3301
	Unaffected side	p-value = 0.489	p-value = 0.8172	p-value = 0.6493

A p-value > 0.05 indicates that the distribution of the corresponding variable did not significantly deviate from normality, supporting the use of parametric statistical methods in subsequent analyses.

2. Pseudo code of the experiment

a. Two-dimensional angle calculation

```
function compute_joint_angle(pointA, pointB, pointC):
    vector1 = pointA - pointB
    vector2 = pointC - pointB

    angle = arccos(dot(vector1, vector2) / (norm(vector1) * norm(vector2)))
    return degrees(angle)
```

b. Three-dimensional angle calculation

```
function compute_projected_angle(pointA, pointB, pointC, midline1,
midline2, midline3):
    u = pointA - pointB
    v = pointC - pointB
    n = cross(midline1 - midline2, midline3 - midline2)
    proj_u = u - projection of u onto n
    proj_v = v - projection of v onto n
    angle = arccos(dot(proj_u, proj_v) / (norm(proj_u) * norm(proj_v)))
    return degrees(angle)
```

c. Calculation of pendulum parameters

```
function compute_pendulum_parameters(filtered_angle_sequence,
begin_angle, rest_angle):
    threshold = rest_angle

    a = first local max
    b = first local min
    c = second local min

    A0 = begin_angle - threshold
    A1 = begin_angle - b
    A2 = a - b
    A3 = a - threshold
    A4 = a - c

    p1 = A1 / (1.6 * A0)
    p2 = A3
    p3 = A4 / (1.6 * A3)

    return {
        'p1': p1,
        'p2': p2,
        'p3': p3
```

}

d. Savitzky–Golay filter implementation

To enhance the quality of the knee angle data, we implemented a third-order Savitzky–Golay filter for signal smoothing. This filter effectively reduced noise from minor body movements and mitigated measurement fluctuations while preserving key waveform characteristics, particularly the peaks and troughs essential for parameter identification.

We use the code *"scipy.signal.savgol_filter(y,7,3, mode= 'nearest')"* to implement the Savitzky–Golay filter, whose window size is 7 and it is third-order.

3. Python packages and version

Pyyaml 5.2
Scipy 1.13.1
Numpy 1.24.3
opencv-python 4.9.0.80
torch 2.5.1+cu121
torchvision
tqdm 4.66.4
cython
cython-bbox
iPython 8.18.1
PyWavelets
pandas
matplotlib
